# Supplementary material for: Are carbon emissions trading and green financial instruments synergistic? -Comprehensive quantitative research based on content analysis
Source: PLoS One. 2024 Mar 7;19(3):e0298601. doi: 10.1371/journal.pone.0298601 (PMC10919601; doi:10.1371/journal.pone.0298601)
Supplement: S1 Data — (ZIP) [file pone.0298601.s001.zip › Data/地级市Z_F工作报告.zip!/║┌┴·╜¡/─╡╡ñ╜¡╩╨/└┤╫╘╣1⁄2╓┌║┼í╛┬φ┐╦╩2╛▌═°í┐_2010.docx]

各位代表：

　　我代表牡丹江市第十四届人大常委会，向大会报告工作，请予审议，并请列席会议人员提出意见。

　　2009年主要工作

　　过去一年，我们在市委的领导下，坚持以科学发展观为统领，紧紧围绕全市工作大局和人民群众切身利益，解放思想，改革创新，依法履行职责，充分发挥地方国家权力机关的作用，听取审议市人民政府专题工作报告22项，听取市人大专委会审议报告11项，交办审议意见45条；对9部法律法规贯彻实施情况开展了执法检查，对28项工作进行了视察、调查，提出建议意见156条；作出决议、决定6项；补选省人大代表1人，任免国家机关工作人员22人(33职)。全面完成了市十四届人大三次会议确定的各项任务，为促进我市经济社会实现追赶型跨越式发展做出了积极贡献。

　　一、围绕中心，推进市委重大决策的贯彻落实

　　2009年，我们紧紧围绕市委提出的工业立市、金融强市和贸旅牵动战略，采取多种方式，全力推进。

　　为应对国际金融危机，推进工业立市战略，结合我市提出的应危机、促发展的十项措施，对桦林轮胎、恒丰纸业、富通空调等20多家骨干企业进行了视察和调研，加强了对工业经济、大项目建设、招商引资等重点工作的监督，针对存在的问题，提出意见和建议，帮助企业和相关部门破解难题保开工、抢抓机遇上项目、对接合作拓市场、改制重组活存量，促进工业经济呈现出稳步上升的良好态势。为保证“双六”目标的实现，我们加强了对计划、预算的日常监测，将监督关口前移，及时了解、掌握全市经济运行总体态势。听取审议了市政府关于2009年市本级预算调整方案情况的报告，并作出了决议；加强了对经济运行的跟踪和预算执行情况的审查，听取审议了市政府财政预(决)算执行情况的报告、审计工作报告及整改报告。

　　为落实金融强市战略和保增长、保民生、保稳定及多渠道融资的要求，听取审议了市政府关于发行信托产品融资的报告，作出了决定，确保企业增加工业产值15亿元以上，增加税收2亿元以上，增加就业岗位上千个，去年上半年投资者拿到了5%的投资回报。在全市深入开展整治金融秩序攻坚战中，为金融单位收回贷款243万元，对不履行还贷义务的18名市、县人大代表，依法做出了处理。

　　为全面实施贸旅牵动战略，加快旅游产业发展，推进旅游产业提档升级，先后两次组织部分驻牡全国、省、市人大代表深入旅游景区(点)和有关单位，对我市旅游业发展情况进行专题视察和调研，并听取审议了市政府贯彻实施《黑龙江省旅游管理条例》和落实《关于突出特色，推进旅游产业发展》(1号议案)情况的报告，提出了加大资源整合力度、加快基础设施建设步伐、优化产业布局和管理体制、提高旅游服务质量等意见，促进了我市旅游业的发展。

　　围绕贯彻落实市委关于公路建设“三年决战”的要求，组织人大代表对市政府开展公路及基础设施建设情况进行了视察和审议，针对存在的问题，提出加强公路交通网络、道路基础设施的监督管理，加快客运枢纽建设等建议。针对牡丹江市区出租汽车停运问题，按照市委的要求，召开有关会议，统一思想、统一步调、提出要求，又专门召开规范性文件备案审查工作领导小组会议，对市政府拟出台的市区出租汽车管理办法进行审查，并提出意见和建议，确保办法顺利出台。一年来，对市政府报送的22件规范性文件进行了备案审查，维护了公民、法人和其它组织的合法权益。

　　二、关注民生，维护人民群众的根本利益

　　常委会把坚持以人为本，改善民生作为行使监督职权、开展工作的出发点和落脚点。为了贯彻落实“中共中央关于推进农村改革发展若干重大问题的决议”，组织三级人大代表，深入县(市)区对新农村建设情况进行了视察，听取审议了市政府关于全市新农村建设中“五清五改五化”及公共服务体系建设情况的报告，并开展了专项工作评议，加快了我市新农村建设的步伐。有关专委会对农民工返乡就业、食用菌产业化发展、新型农村合作医疗等工作进行了调查。为了促进社会保障工作，组织人大代表对全市社会保障工作进行了视察和审议，针对存在的问题，提出加大宣传力度、加强调查研究、加大资金投入、加强队伍建设等意见，市政府认真研究落实，对医保、失业和养老金发放标准分别提高了16.5%、11.5%和14.7%。

　　为了确保市十四届人大一次会议确定的关于加快棚户区改造(1号议案)扎实推进，组织人大代表对棚户区改造和廉租房建设情况进行了视察和审议，首次邀请10名市民代表旁听了常委会会议，增强了常委会议事、决策的透明度，自觉接受人民群众的监督；常委会跟踪监督了市十四届人大二次会议确定的关于“一河三溪六湖”治理(1号议案)进展情况，经常与市政府有关部门沟通联系，提出建议意见，实现了截污主管线2.94公里，清淤6万立方米，完成投资2976万元。通过开展以“加强农村环境保护，提高农民环保意识”为主题的“龙江环保世纪行”活动，督促解决了个别村屯因在江堤内耕地施作、生产生活污水排入江中等问题，避免了对水源地的污染。

　　为了推进有关法律法规的落实，听取审议了市政府贯彻实施老年人权益保障法、残疾人保障法、妇女权益保障法情况的报告，督促解决了妇儿工作经费、老龄工作机构、婚前身体检查等方面存在的问题；有关专委会对贯彻实施体育法、科技成果转化法、食品安全法、粮食流通管理条例、全国人大关于司法鉴定管理问题的决定等法律法规情况进行了执法检查，对全市法院清理民事执行积案工作、涉法涉诉问题加大了监督力度，对受理的47件涉法涉诉案件基本做到了件件有结果。

　　高度重视人大信访工作，为人民群众排忧解难，受理接待群众来信来访532件(次)，督促解决了一批城镇房屋拆迁、农村土地纠纷等疑难信访问题。市人大信访办被市委、市政府授予“重视支持信访工作特殊贡献奖”。

　　三、拓展平台，充分发挥代表作用

　　为了提高代表的履职能力，采取多种形式加强代表培训工作。去年7月，我们组织120名人大代表和机关干部参加了全国地方人大干部履职培训班，集中学习了人大工作的必备知识；邀请专家对常委会组成人员和机关干部进行金融知识等方面的专题讲座，提高了素质，增长了才干；为基层人大代表和机关干部作了法律专题辅导。

　　为了更好地保障人大代表依法履职，在开展“三个一”(即联系一个单位、做一件实事、提出一条建议)和“十百千万”(即捐建十条街路、认养十片绿地、保护百年古树、栽植千棵市树--云杉、万株市花--牡丹)活动的基础上，开展了“履职尽责为牡丹江经济社会发展做贡献”活动，四城区人大常委会积极组织人大代表集资超额完成了整修街路任务并完善了古榆苑工程，为城市建设做出了贡献。为充分发挥代表小组作用，制定下发了代表小组活动意见，科学合理地划分了23个代表活动小组，明确任务、提出要求，为代表活动提供了保障。这些小组围绕打造沿边开放先导区、哈牡绥东对俄贸易加工区、铁路运输、社区医疗卫生、城市居民最低生活保障等工作开展了视察活动，提出建议意见，涌现出一批优秀代表和先进代表小组。

　　坚持走访联系代表制度，常委会领导深入县(市)区走访联系了部分省、市、县三级人大代表，面对面征求代表意见建议，帮助解决困难和问题，为代表履行职责创造条件。坚持把办理代表建议作为督促有关部门改进工作、解决群众反映的热点难点问题的重要渠道，对市十四届人大三次会议代表提出的153件建议，采取上门督办、重点督办、全程跟踪、听取汇报等方法，确保代表建议办理质量，代表建议满意和基本满意率达98%以上。

　　四、夯实基础，提高履职能力

　　按照市委统一部署，认真开展了深入学习实践科学发展观活动，市人大党组高度重视，加强领导，精心组织，周密部署，紧紧围绕“提高履职能力，推动科学发展”这一主题，研究部署学习实践活动，共组织学习讨论20次，学习辅导16次，召开座谈会7次，撰写调研报告14篇，征求各方面的意见建议69条，提出整改措施29项。通过深入学习讨论，强化理论武装，突出实践特色，加强调查研究，广泛听取意见，坚持边学边改，增强了贯彻落实科学发展观的自觉性。根据形势发展的需要，对常委会和机关的有关制度、规则进行了修改完善；通过努力建设学习型机关、推行工作目标责任制、转变工作作风，加强了常委会和机关建设。

　　针对人大工作面临的新形势、新任务，充分利用各级新闻媒体和人大刊物、网站，大力宣传人民代表大会制度、民主法制建设、依法履行职责等情况，在国家、省、市新闻媒体发表稿件209篇，有4篇新闻稿件在省人大好新闻评比中获奖。为了扩大对外交往，先后接待了俄罗斯、韩国地方议会代表团，加强了多方面的交流与合作。通过召开县(市)区人大主任座谈会、出席全国二十城市人大工作研讨会、外出考察调研，学习了经验，加强了联系，推动了工作。

　　常委会注重发挥专门委员会的作用，经常与各专门委员会沟通情况，交换意见，统一思想；各专门委员会紧紧围绕市人大常委会的中心任务，做了大量卓有成效的工作，有力地促进了常委会整体工作水平的提高。

　　各位代表，市十四届人大常委会一年来所取得的成绩，是市委正确领导的结果，是全体人大代表、常委会组成人员、专委会委员及机关工作人员共同努力的结果，也是“一府两院”、县(市)区人大常委会和广大人民群众大力支持的结果。在此，我代表市人大常委会，向大家表示衷心的感谢!

　　常委会一年来的工作虽然取得了一些成绩，但按照市委要求和全市人民的期待，还存在一些需要改进的地方。一是在视察和审议中，要改进方式方法，进一步提高视察质量和审议实效；二是要进一步加强学习培训，提高服务保障水平，更好地发挥代表作用；三是要进一步探索创新，更好的履行职责，提高人大工作整体水平。

　　2010年主要任务

　　今年，是“十一五”收尾之年，也是“超千破百”的起步之年，更是我们正视困难，面对挑战，加快经济发展方式转变，推进经济转型升级的重要一年。今年常委会工作的总体要求是：深入贯彻落实党的十七届四中全会和市委十届十次全委(扩大)会议精神，以科学发展观为统领，在市委的领导下，紧紧围绕“超千破百”的目标，坚持务实创新，突出工作重点，增强履职实效，充分发挥地方国家权力机关的作用，大力加强民主法制建设，为实现新一轮追赶型跨越式发展做出新的贡献。

　　一、依法行使职权，推动追赶跨越发展

　　要紧紧围绕全市中心工作，围绕加快发展，围绕“超千破百”目标，找准人大工作的切入点。依法行使监督权。进一步加强和改进监督工作，把监督与支持有机结合起来，围绕发展进行监督，通过监督促进发展。围绕市委决策部署，听取审议市政府关于工业立市战略实施情况和企业转型升级情况的报告；以推进公共财政建设为重点，听取审议市政府上半年财政预算执行情况的报告，上年度财政决算报告、审计工作报告及整改报告，开展上半年国民经济和社会发展计划执行情况的视察、检查，对全市财源建设情况进行调查；大力推进沿边开放先导区建设，听取审议市政府关于开发区建设情况的报告，促进哈牡绥东贸易加工区省级战略的顺利实施；为强力推进“旅游名镇”建设步伐，对市政府落实2009年市十四届人大三次会议确定的“1号议案”进行跟踪检查，促进旅游业快速发展；围绕生态资源环境保护工作，听取审议市政府开展植树绿化和落实《牡丹江市城市绿地水域保护管理暂行办法》情况的报告；为完善城市发展格局，提高城市品位，打造精品城市，改善人居环境，听取审议市政府关于贯彻实施城乡规划法情况的报告，对市政府《关于2009年城市建设计划执行情况和2010年城市建设计划安排意见的报告》进行审查，继续深入开展“龙江环保世纪行”活动，配合省人大常委会做好《镜泊湖风景名胜区保护管理条例》、《牡丹峰自然保护区管理条例》立法前的各项基础工作。依法行使重大事项决定权。要从改革发展稳定的大局出发，依法审议、决定带有全局性、长远性、根本性的重大问题以及人民群众普遍关心的热点、难点问题，依法适时作出决议、决定，更好地维护人民群众的根本利益，为党委的重大决策科学化、民主化提供重要保证。依法行使人事任免权。坚持人大依法任免与党管干部相统一，认真贯彻落实中央和省、市委关于选拔任用干部工作的有关规定，对提请市人大常委会审议任命的国家机关工作人员，要坚持任前考察，认真听取常委会组成人员的审议意见，充分发扬民主，搞好任中监督，通过采取专项工作评议、听取专项工作报告等办法，加强任后监督，确保被任命的国家机关工作人员依法行政、公正司法。

　　二、坚持以人为本，贴近人民群众需求开展工作

　　要把人民群众普遍关注的热点、难点问题作为人大工作的重点，通过依法行使职权，牢固树立群众观念，保持与人民群众经常性的联系，及时体察民情、反映民意，推动解决人民群众最关心、最直接、最现实的利益问题。今年，常委会要听取审议市政府关于贯彻实施工会法、全民健身条例、宗教事务条例情况的报告，开展外向型农业发展、民族文化产业、民族教育情况的调查；围绕促进司法行政机关公正执法、维稳措施落实，加大监督和支持力度，听取审议市中级人民法院关于民商事案件执行工作情况的报告，开展对市人民检察院控申工作、市人民政府打击经济犯罪工作的检查；围绕保障人民群众的身心健康，开展贯彻实施食品安全法以及清真食品经营管理情况的调查；为进一步促进“三农”工作，开展农民专业合作社法、农田水利条例实施情况的检查；针对人民群众普遍关注、反映比较集中的问题，开展物价法、邮政法、建筑工程质量条例、广播电视管理条例实施情况的执法检查。认真接待受理人民群众来信来访，及时督办和转办信访事项，把人大信访与人大依法监督结合起来，努力化解矛盾纠纷，推进社会和谐稳定。

　　三、积极探索创新，促进人大工作迈出新步伐

　　要以贯彻落实监督法为重点，改进视察审议的方式方法，坚持随机抽查与重点检查相结合，防止和克服形式主义，提高审议质量，增强监督实效；听取审议专项工作报告要坚持会前组织视察，会中分合审议、会后跟踪问效制度，对重点难点问题进行反复督查，对带有普遍性的问题采取上下联动监督。要把听取审议专项工作报告与执法检查结合起来，把人大监督和群众监督、舆论监督结合起来，听取审议的报告及审议意见、“一府两院”对审议意见整改情况及时向代表通报并向社会公布。要进一步探索开展专项工作评议的方式方法，坚持从实际出发，修改完善专项工作评议暂行办法，组织各级人大代表对全市大项目建设情况进行集中视察，并开展专项工作评议。要加强规范性文件备案审查工作，争取设立备案审查的专门机构，完善备案审查机制，强化备案审查的监督管理，做到及时审查和备案。围绕发展社会主义民主政治，扩大公民有序政治参与，积极探索公民旁听常委会会议的形式，根据会议议题，有选择性地开放公民旁听常委会会议，使广大群众了解人大常委会履行职责情况。以“创新人大工作方式，发挥人大履职效力”为主题，召开一次宪法和人大建设理论研讨会，提高理论研究水平。

　　要以贯彻落实代表法、组织法和选举法为重点，举办一期高层次、大规模的培训班，全面提高代表的理论水平、政治素养和履职能力；采取以会代训的形式，召开一次如何当好人大代表的经验交流会，表彰优秀代表和先进代表小组；改进代表活动的方式方法，继续推进“三个一”和“走访联系代表”活动，建立健全代表联系选民、代表列席常委会会议、开展“三查(察)”活动的长效机制，恢复代表热线电话，开展形式多样的代表小组活动；组织代表广泛开展“为超千破百做贡献活动”，充分发挥代表在追赶跨越发展中的作用；加大为代表提出议案建议的服务力度，创新督办方式，变普遍督办为重点督办，提高代表对建议办理的满意率；加大代表履职考核力度，积极开展代表述职活动，营造争先创优的良好氛围。

　　要为常委会行使职权做好服务工作，及时将有关报告、参阅文件发给组成人员，为其了解掌握有关情况提供依据；认真整理常委会审议意见，转交“一府两院”办理；加强与主流媒体的沟通，为其宣传人大工作创造条件；办好《牡丹江人大》刊物和门户网站。同时，要密切与县(市)区人大常委会的工作联系，加强工作指导；密切与国内兄弟地市人大常委会的交流，学习借鉴经验；加强与外国友好城市议会交往，巩固和完善交流机制，促进多个领域的交流与合作。

　　四、加强自身建设，为常委会行使职权提供有力保障

　　坚持正确的政治方向。要把思想政治建设放在首位，自觉接受市委的领导，定期向市委请示汇报工作，积极参加市委组织的重大活动，认真完成市委交办的各项任务，保证市委的决策部署在人大常委会得到贯彻落实。加强常委会自身建设。继续完善常委会有关制度规定，推动工作规范化、制度化，提高常委会会议质量、发挥组成人员作用；大力改进工作作风，坚持求真务实，大兴调查研究之风，经常深入基层、深入群众、深入实际，体察民情，反映民意，集中民智，使常委会作出的决议决定、提出的审议意见更加贴近经济社会实际，贴近人民群众需要。加强机关建设。进一步完善并严格执行机关内部管理制度，理顺关系、规范程序、明确责任、提高效率。大力推行工作目标责任制，努力营造勤于学习的进取氛围，保持昂扬向上的精神状态，发扬求真务实的工作作风，塑造清正廉洁的良好形象。切实加强机关干部的学习培训，提高政治素质和业务能力；加强干部的培养和交流，优化干部队伍结构，坚持以提高工作质量和效率为重点，狠抓工作落实，努力提高机关工作水平。

　　各位代表，牡丹江正处在大发展、快发展的关键时期，崇高的使命激励着我们，艰巨的任务鞭策着我们。让我们在市委的领导下，解放思想，务实创新，锐意进取，埋头苦干，以对党和人民高度负责的精神，忠实履行神圣职责，为实现新一轮追赶型跨越式发展做出更大的贡献！
